# *In vitro* and *in vivo* multidrug resistance reversal activity by a Betti-base derivative of tylosin

**DOI:** 10.1038/sj.bjc.6605716

**Published:** 2010-06-15

**Authors:** N Gyémánt, H Engi, Z Schelz, I Szatmári, D Tóth, F Fülöp, J Molnár, P A M de Witte

**Affiliations:** 1 Faculty of Medicine, Institute of Medical Microbiology and Immunobiology, University of Szeged, Dóm tér 10, H-6720 Szeged, Hungary; 2 Faculty of Pharmaceutical Sciences, Laboratory for Pharmaceutical Biology, K.U.Leuven, O&N PB 824, Herestraat 49, B-3000 Leuven, Belgium; 3 Department of Pharmaceutical Chemistry, Faculty of Pharmacy, University of Szeged, Zrínyi u.9, H-6720 Szeged, Hungary

**Keywords:** P-glycoprotein, resistance, doxorubicin, tumour, Betti-base, tylosin

## Abstract

**Background:**

The multidrug resistance (MDR) proteins are present in a majority of human tumours. Their activity is important to understand the chemotherapeutic failure. A search for MDR-reversing compounds was conducted among various Betti-base derivatives of tylosin.

**Methods:**

Here, we evaluate the *in vitro* and *in vivo* P-glycoprotein (P-gp)-modulating activity of the most promising compound *N*-tylosil-1-*α*-amino-(3-bromophenyl)-methyl-2-naphthol (TBN) using human MDR1 gene-transfected and parental L5178 mouse lymphoma cell lines.

**Results:**

*In vitro* experiments showed that TBN dramatically increased the P-gp-mediated cellular uptake of the fluorescent substrate rhodamine 123. Similarly, TBN was found to act as a very potent enhancer of the cytotoxicity of doxorubicin on the resistant cell line. We also provide *in vivo* evidence using DBA/2 mice in support for an increased tumoural accumulation of doxorubicin, without affecting its tissue distribution, resulting in an enhanced antitumoural effect.

**Conclusion:**

Our results suggest that TBN is a potent modulator of the P-gp membrane pump and that the compound could be of clinical relevance to improve the efficacy of chemotherapy in MDR cancers.

As tumour cells may acquire cross-resistance to many structurally and functionally unrelated anticancer drugs, development of multidrug resistance (MDR) is one of the main reasons for treatment failure in the chemotherapy of malignant tumours. A common cause of MDR is expression of the MDR gene (mdr1) and its protein product P-glycoprotein (P-gp), a membrane pump endowed with a drug-efflux activity. Consequently, by means of inhibition of the P-gp pump, thus reversing cellular resistance, one should be able to restore the inherent potency of antitumour agents. In the past decades, a quest for effective MDR reverting drugs has emerged, and numerous potent P-gp inhibitors have been tested so far, including verapamil, cyclosporine analogues (Krishna and Mayer, 2000) and the anthranilamide derivative tariquidar (Roe *et al*, 1999). Unfortunately, to date, there is no resistance modifier in medical practice; however, encouraging clinical trials were reported for tariquidar (Pusztai *et al*, 2005) and another specific P-gp inhibitor ONT-093 (Chi *et al*, 2005).

To find novel effective compounds with MDR reversal activity, more than a hundred compounds containing a carbo- and/or heterocyclic skeleton and various functional groups, prepared in the recent years in the Institute of Pharmaceutical Chemistry, University of Szeged, were tested. As a result of the outcome of our screening, a large set of Betti-base derivatives was prepared and each of the compounds further examined for their *in vitro* P-gp inhibitory effects. Of all compounds investigated, the most promising was *N*-tylosil-1-*α*-amino-(3-bromophenyl)-methyl-2-naphthol (TBN), a 1-*α*-amino-(3-bromophenyl)-methyl-2-naphthol derivative of tylosin (see Figure 1). Tylosin is a compound belonging to the macrolides, a class of antibiotics, which are clinically used and are considered to be safe with minimal side effects. Betti-bases can easily be prepared by the hydrolysis of the condensation product obtained from the corresponding aromatic aldehyde with 2-naphthol in the presence of ammonia (Betti, 1941; Szatmári *et al*, 2003; Szatmári and Fülöp, 2004). Betti-bases featuring a free primary amine readily react with the aldehyde group present in tylosin. Under reductive amination conditions, the Schiff base formed is further stabilised by reduction (Omura, 1984).

This study was performed to fully appreciate the *in vitro* and *in vivo* MDR reversal activity of the compound. We show that TBN, and much less so the individual Betti-base and tylosin moiety, is able to dramatically increase the cellular uptake of the fluorescent P-gp substrate rhodamine 123 in human MDR1 gene-transfected mouse T-cell lymphoma drug-resistant cell line L5178Y and to fully reverse the cellular resistance against doxorubicin. We also provide *in vivo* evidence using DBA/2 mice bearing syngeneic L5178Y tumours in support for an increased tumoural accumulation of doxorubicin, without affecting its tissue distribution, resulting in an enhanced antitumoural effect. Our results, therefore, suggest that TBN could be of clinical relevance to improve the efficacy of chemotherapy in MDR cancers.

## Materials and methods

### Chemistry

#### Experimental section

Melting points were determined on a Kofler micro-melting apparatus and are uncorrected. Elemental analyses were performed with a Perkin-Elmer 2400 CHNS elemental analyser (Perkin-Elmer, Waltham, MA, USA). Merck Kieselgel 60F_254_ plates were used for thin layer chromatography.

#### Materials

**Figure 1:**
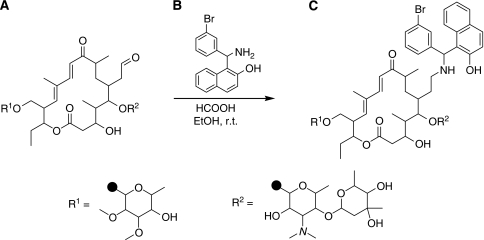
Reaction scheme. (**A**) Tylosin, (**B**) Betti-base, (**C**) TBN (*N*-tylosil-1-*α*-amino-(3-bromophenyl)-methyl-2-naphthol). r.t., room temperature.

TBN was prepared (see Figure 1) according to Szatmári *et al* (2003) by stirring a solution of tylosin tartrate (Sigma, St Louis, MO, USA) (0.20 g, 0.18 mmol), the Betti-base (1-*α*-amino-(3-bromophenyl)-methyl-2-naphthol) (0.2 mmol) and formic acid (0.01 g, 0.22 mmol) in ethanol (20 ml) at room temperature for 24 h. After removal of the solvent under reduced pressure, the residue was taken up in aqueous Na_2_CO_3_ solution (10 ml) and extracted three times with chloroform (10 ml). The organic layer was collected, dried with sodium sulphate and then the solvent was evaporated. The crude product was crystallised with *n*-hexane (5 ml) and recrystallised from *n*-hexane–diisopropyl ether (5 : 2).

The yield was 0.18 g (81%) and melting point 160–162°C. Analytically calculated for C_63_H_91_BrN_2_O_17_ was C, 61.60; H, 7.47; N, 2.28 and the results were C, 61.72; H, 7.41; N, 2.30.

### Bioassays

#### Chemicals

Doxorubicin HCl was purchased from Teva Pharma (Wilrijk, Belgium) and Aventis Pharma (Brussels, Belgium), respectively. Other compounds used were rhodamine 123 (Sigma), verapamil (EGIS, Hungarian Pharmaceutical Company, Budapest, Hungary), MTT (thiazolyl blue, Sigma), perchloric acid (Sigma-Aldrich, Steinheim, Germany) for acidified water (pH 2.05) and sodium dodecyl sulphate (SDS) (Sigma). Water was purified by the Milli-Q system (Millipore, Milford, USA).

#### Cell culture and animals

L5178 mouse T-cell lymphoma cells were transfected with pHa MDR1/A retrovirus, as described earlier (Cornwell *et al*, 1987). The MDR1-expressing cell lines were selected by culturing the cells in the presence of 60 ng ml^–1^ colchicine. The parent L5178 mouse T-cell lymphoma cells and the transfected subline were cultured in McCoy's 5A medium supplemented with 10% heat-inactivated horse serum L-glutamine and antibiotics. The cell lines were incubated in a humified atmosphere (5% CO_2_, 95% air) at 37°C. The DBA/2 inbred mice (female, 5–7 weeks old) and Balb/c mice (female, 5–7 weeks old) were obtained from Charles River Laboratory (France). After an experiment, the animals were killed by cervical dislocation. All aspects of the animal experiment and husbandry were carried out in compliance with national and European regulations and were approved by the Animal Care and Use Committee of K.U.Leuven.

#### Flow cytometric assay of rhodamine 123 cellular accumulation

Parental or transfected L5178 cells were adjusted to a density of 2 × 10^6^ per ml, resuspended in serum-free medium and distributed as 0.5-ml aliquots into microvials. Compounds at different concentrations (verapamil (control), TBN, the corresponding Betti-base moiety and tylosin) or vehicle were added and the samples incubated for 10 min at room temperature. Next, 10 *μ*l (5.2 *μ*M final concentration) of a solution of rhodamine 123 (Sigma) was added and the cells were incubated for a further 20 min at 37°C, washed twice and resuspended in 0.5 ml PBS for analysis. The fluorescence of the cell population was measured with a Beckton Dickinson FACScan flow cytometer. The percentage mean fluorescence intensity was calculated for the parental and transfected L5178 cells, and compared with the untreated cells. A fluorescence activity ratio (FAR) was calculated from the following equation on the basis of the measured fluorescence values: 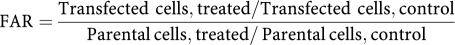


#### *In vitro* antiproliferative assay

Parental or transfected L5178 cells were treated with different concentrations of TBN, the corresponding Betti-base, tylosin or doxorubicin, or combinations of different concentrations of doxorubicin with two fixed concentrations of TBN, the Betti-base or tylosin, or vehicle to investigate the antiproliferative effect of the compounds or their combination on the cells. First, the compounds were diluted in a volume of 100 *μ*l medium in each well, then 1 × 10^4^ cells in 50 *μ*l of medium were added. The culture plates were further incubated at 37°C for 72 h. At the end of the incubation period, 20 *μ*l of MTT solution (5 mg ml^–1^) was added to each well. After incubation at 37°C for 4 h, 100 *μ*l of SDS solution (10%) was added and the plates were further incubated at 37°C overnight. The relative cell density was determined by measuring the optical density (OD) at 550 nm (reference 630 nm) with a Dynatech MRX vertical beam ELISA reader. Inhibition of cell growth (as a percentage) was determined according to the formula: 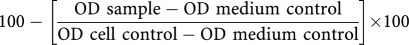


#### Short-term cellular accumulation and cytotoxicity of doxorubicin

Parental or transfected L5178 cells were plated using 24-well plates (4 × 10^6^ cells per 1.5 ml per well) in serum-free medium; TBN (10 *μ*M) was added (or not) 30 min before exposing the cells to 40 *μ*M doxorubicin at 37°C for 1 h. For the toxicity assay, the cell suspensions were centrifuged on 4500 r.p.m. for 5 min and washed twice in serum-free medium. The cells were cultured for 48 h at 37°C using 96-well plates (10^5^ cell per 0.15 ml per well) in serum-supplemented medium. Cell proliferation was evaluated by the above-mentioned MTT test. For the accumulation assay, another part of the cell suspensions was washed twice with ice-cold PBS. After resuspending in water, cells were extracted and the amount of doxorubicin quantified by liquid chromatography (LC) (see further). The results were calculated assuming a mean volume of 3 *μ*l per 10^6^ cells (Lin *et al*, 1991).

#### Tumoural accumulation of doxorubicin

The DBA/2 mice were anesthetised and injected subcutaneously with 4 × 10^6^ cells of the parental or transfected L5178 cells. Tumours were allowed to grow to ca. 0.5 cm diameter, after which the animals were treated once with TBN (or vehicle) administered either intraperitoneally (i.p.) (10 or 50 mg kg^–1^) or intravenously (i.v.) (10 mg kg^–1^) through a lateral tail vein, and doxorubicin (i.p. or i.v., 10 mg kg^–1^) or vehicle. Tumours were excised 24 h later and stored at −20°C until extraction and LC analysis.

#### *In vivo* pharmacokinetic study of doxorubicin

The TBN (50 mg kg^–1^) or vehicle was administered i.p. 3 h before the i.v. administration of doxorubicin (10 mg kg^–1^) or vehicle to Balb/c mice. At various time points (30 min, 1, 5, 24 and 48 h) after doxorubicin injection, mice were killed. Plasma and tissue samples from liver, kidneys and heart were collected and stored at −20°C until extraction and LC analysis.

#### Sample extraction and doxorubicin quantification

The amount of doxorubicin in plasma and tissues was quantified as described by van Asperen *et al* (1998). The LC system consisted of a Hitachi Elite LaChrom L-2130 solvent delivery module and a Hitachi Elite LaChrom L-2480 fluorescence detector (Hitachi High-Technologies Corporation Tokyo, Japan). The LiChroCART 250–4 analytical column packed with 5 *μ*m Purospher STAR material (Merck, Darmstadt, Germany) was protected by a LiChrospher guard column (4 × 4 mm) (Merck, Darmstadt, Germany). The mobile phase was composed of acidified water (pH 2.05)/acetonitrile/tetrahydrofuran (64 : 35 : 1, v/v/v) and was degassed by ultrasonication. A flow rate of 0.8 ml min^–1^ was used. The column eluent was monitored fluorimetrically at 460 nm (ex) and 550 nm (em).

#### *In vivo* efficacy test

The ability of TBN to potentiate the antitumour activity of doxorubicin was evaluated using the MDR1 gene-transfected L5178 xenografts. When the tumour size reached a diameter of ca. 0.5 cm, the animals were randomised and treated every second day with TBN (10 or 50 mg kg^–1^) or vehicle that was administered i.p. 3 h before doxorubicin (i.p., 2 or 4 mg kg^–1^). Animals were weighed and the tumours measured every second day. The tumour volume (TV) was calculated from the following equation (according to Zalatnai and Molnar, 2006): 
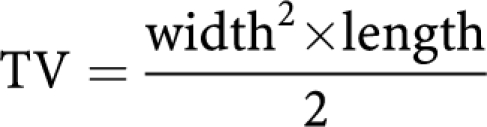


Animals were killed and tumours were excised and weighed on 12 day.

#### Statistics

Analysis of variance or Student's *t-*test using Prism 5 (Graphpad software) was performed to determine the significance of differences between the means. Significance was accepted at *P*<0.05.

## Results

### Flow cytometric assay of rhodamine 123 accumulation in tumour cells

The TBN and its two building blocks, tylosin and the corresponding Betti-base, were examined in a short-term rhodamine 123 (R123) accumulation assay for their P-gp inhibitory effect, using both the parental and human MDR1 gene-transfected L5178 mouse lymphoma cells. The results are displayed in Table 1. Compared with untreated resistant cells, the presence of TBN resulted in a ca. 100-fold higher R123 accumulation in mouse lymphoma cells, even at a low concentration, whereas the tylosin and the Betti-base showed only limited effect. In these short-term experiments, no signs of toxicity or cell damage were observed.

### *In vitro* antiproliferative effects

Using an antiproliferative assay, the cytotoxic effects of the three compounds and doxorubicin on the parental and transfected L5178 cells were examined. From the inhibitory curves obtained, corresponding IC_10_, IC_50_ and IC_90_ values were calculated (Table 2). The Betti-base and TBN displayed a somewhat similar antiproliferative profile, at least in case of the transfected cells, whereas the two cell lines used exhibited a much lower sensitivity towards tylosin. Of interest, depending on the IC value under investigation, the parental cells were about four-fold (IC_10_), 40-fold (IC_90_) or 80-fold (IC_50_) more sensitive towards doxorubicin as compared with the transfected L5178 cells.

Consequently, the ability of TBN to reverse the resistance against doxorubicin of the transfected cells was investigated and its was used at its IC_10_ concentration, that is 9.4 *μ*M and a 10-fold lower concentration (IC_10/10_), that is 0.94 *μ*M, in combination with different concentrations of doxorubicin. At both concentrations, TBN enhanced significantly the cytotoxic effect of doxorubicin on the resistant L5178 cells (Table 3). Similar experiments showed much less effects for the Betti-base and tylosin.

### Short-term *in vitro* accumulation and cytotoxicity of doxorubicin

**Figure 2:**
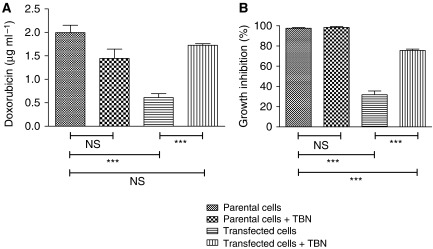
(**A**) Doxorubicin accumulation (in *μ*g ml^–1^, assuming an average volume of 3 *μ*l per 10^6^ cells) in non-resistant (parental cells) and human MDR1 gene-transfected (transfected cells) L5178 mouse lymphoma cells. Cells were exposed to 40 *μ*M doxorubicin for 1 h (with or without 10 *μ*M TBN), after which they were extracted and the amount of doxorubicin quantified by LC. The values are mean ± s.d. of three independent experiments. (**B**) Effect of 40 *μ*M doxorubicin for 1 h (with or without 10 *μ*M TBN) on the proliferation of the different cells. After treatment, cells were washed and cultured for 48 h at 37°C using 96-well plates. The values are mean ± s.d. of three independent experiments (each conducted in triplicate). Significant differences between means, as specified by capped lines, are indicated by ^***^P<0.0001. ns, not significant.

To test whether the increased cytotoxicity observed in the earlier experiments correlates with an enhanced intracellular concentration of doxorubicin, we exposed the parental and transfected L5178 tumour cells to 40 *μ*M doxorubicin for 1 h in the presence or absence of TBN (10 *μ*M). The amount of doxorubicin that accumulated in non-resistant L5178 cells was about three- to four-fold the one recovered from resistant cells. Treatment by TBN was able to restore the amount of doxorubicin accumulating in the resistant cells (Figure 2A). Altogether, we found evidence that a correlation exist between the accumulation of doxorubicin and its toxic effects (Figure 2B) on L5178 cells *in vitro*, at least after a short exposure of the cells to doxorubicin.

### *In vivo* doxorubicin accumulation

**Figure 3:**
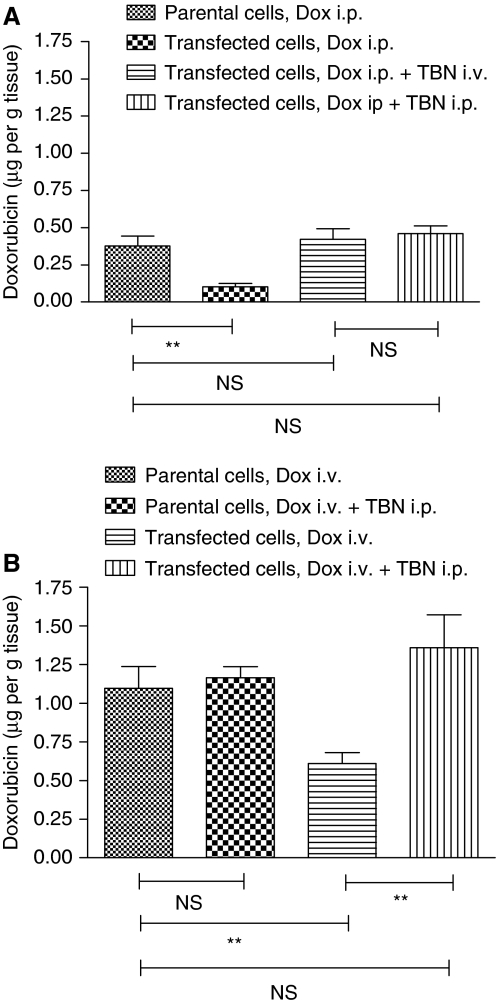
Accumulation of doxorubicin (in *μ*g g^–1^) in tumours consisting of parental or transfected L5178 cells growing subcutaneously in DBA/2 mice. Mice were treated with doxorubicin (**A**: 10 mg kg^–1^, i.p.; **B**: 10 mg kg^–1^, i.v.). (**A**) TBN (10 mg kg^–1^) was administered (or not) i.p. 3 h or i.v. 1 h before doxorubicin treatment; (**B**) TBN (50 mg kg^–1^) was administered (or not) i.p. 3 h before doxorubicin treatment. Tumours were excised 24 h later, stored at −20°C until extraction and LC analysis. The values are mean ± s.d. of seven independent experiments. Significant differences between means, as specified by capped lines, are indicated by ^**^P<0.001. ns, not significant.

When doxorubicin (10 mg kg^–1^) was injected i.p., low levels of doxorubicin were detected in MDR1-expressing L5178 tumours (Figure 3A). In these conditions, TBN (10 mg kg^–1^) was able to enhance the tumoural accumulation of doxorubicin when it was administered i.v. 1 h or i.p. 3 h before doxorubicin to the levels recovered from non-resistant L5178 tumours. However, when TBN and doxorubicin were simultaneously injected, no increase of the doxorubicin could be observed (data not shown). After administering 10 mg kg^–1^ doxorubicin i.v., the recovered amount of drug was more than the double as compared with the one obtained after similar i.p. injections (Figure 3B). As can be seen, also in this case, TBN (i.p., 50 mg kg^–1^) was able to restore the doxorubicin accumulation in MDR1-expressing L5178 tumours.

### *In vivo* pharmacokinetic study of doxorubicin

**Figure 4:**
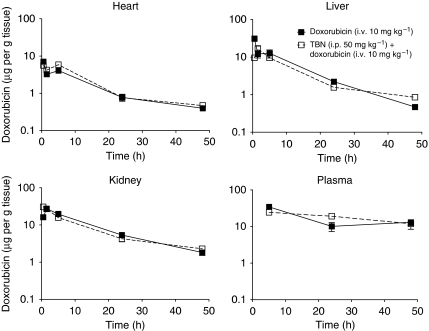
Tissue distribution of doxorubicin. Balb/c mice were injected with doxorubicin alone i.v. (10 mg kg^–1^) or with TBN (50 mg kg^–1^) i.p. 3 h before doxorubicin i.v. (10 mg kg^–1^). At various times after doxorubicin injection (30 min, 1, 5, 24 and 48 h) three to five mice were killed. Plasma and tissue samples from liver, kidneys and heart were collected and stored at −20°C until extraction and liquid chromatography analysis. The values are mean ± s.e.

Administration of TBN at a dose of 50 mg kg^–1^ (i.p.) 3 h before doxorubicin (10 mg kg^–1^, i.v.) in Balb/c mice had no significant effect on the pharmacokinetic behaviour of doxorubicin (Figure 4).

### *In vivo* efficacy evaluation

**Figure 5:**
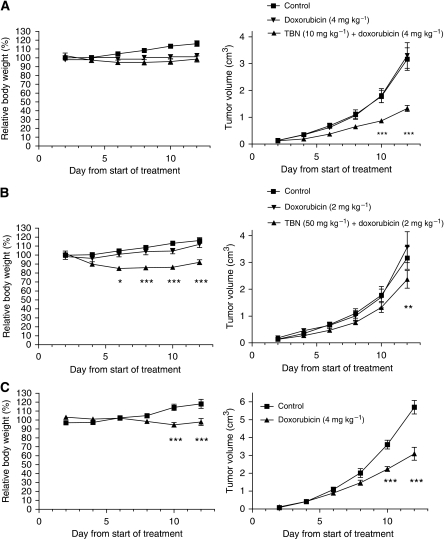
Antitumour activity of doxorubicin in the presence or absence of TBN. Tumours consisted of parental (**C**) or transfected L5178 cells (**A**, **B**) growing subcutaneously in DBA/2 mice. When the tumour size reached 0.5 cm diameter, the animals were randomised and treated every second day. The TBN (**A**: 10 mg kg^–1^; **B**: 50 mg kg^–1^) was administered (or not) i.p. 3 h before doxorubicin (**A**: 4 mg kg^–1^; **B**: 2 mg kg^–1^, **C**: 4 mg kg^–1^) was injected i.p. Animals were weighed every second day and the experiments were terminated on 12th day. The values represent mean ± s.e. of five to eight animals per group. Significant differences on the individual days between means of the ‘doxorubicin’ arm and the ‘doxorubicin ± TBN’ arm (**A**, **B**) or ‘control’ arm and the ‘doxorubicin’ arm (**C**) are indicated by ^*^P<0.01, ^**^P<0.001 and ^***^P<0.0001. No indication implies no significant difference between the means.

**Figure 6:**
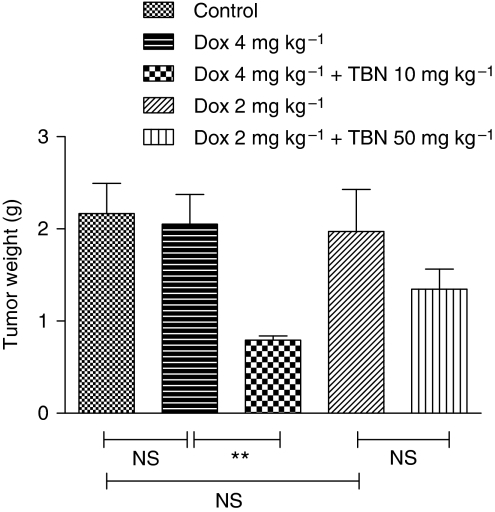
Effect of doxorubicin on the tumour weight in the presence or absence of TBN. Tumours consisted of transfected L5178 cells growing subcutaneously in DBA/2 mice. When the tumour size reached 0.5 cm diameter, the animals were randomised and treated every second day. The TBN (10 or 50 mg kg^–1^) was administered i.p. 3 h before doxorubicin (4 or 2 mg kg^–1^, respectively) was injected i.p. On day 12, animals were killed and tumours were excised and weighed. The values represent mean ± s.e. of five to eight animals per group. Significant differences between means, as specified by capped lines, are indicated by ^**^P<0.001. ns, not significant.

The ability of TBN to enhance the antitumoural efficacy of doxorubicin was tested on DBA/2 mice bearing MDR1-expressing and non-transfected, sensitive L5178 tumours (Figure 5). The growth rate of the MDR1-expressing L5178 tumours was significantly reduced by administration of TBN (10 mg kg^–1^) 3 h before doxorubicin (4 mg kg^–1^) as compared with doxorubicin alone (Figure 5A). In contrast, the other treatment regimen (TBN: 50 mg kg^–1^, doxorubicin: 2 mg kg^–1^) was much less effective (Figure 5B). A similar outcome was obtained by measuring the weight of the tumours excised on day 12 (Figure 6). Obviously, doxorubicin alone had an antitumoural effect on sensitive L5178 tumours (Figure 5C). The combination of 50 mg kg^–1^ TBN and doxorubicin resulted in a slight decrease in body mass of mice as a function of time compared with the condition in which only doxorubicin was used. This was not the case when 10 mg kg^–1^ TBN was used (Figure 5).

## Discussion

This study shows that TBN is a potent modulator of P-gp-mediated MDR, as shown by using both *in vitro* and *in vivo* a human MDR1 gene-transfected mouse lymphoma cell line and its parental doxorubicin-sensitive counterpart. Conversely, by using an *in vitro* accumulation and antiproliferative assay, we were able to reveal that the building blocks of TBN, that is tylosin and the corresponding Betti-base, display rather weak effects on P-gp-mediated MDR. As rhodamine 123 and doxorubicin used in these tests, respectively, are well-known substrates of P-gp (Mankhetkorn *et al*, 1999; Marbeuf-Gueye *et al*, 2000; Loo and Clarke, 2002; Wang *et al*, 2006), our data show that by introducing a nitrogen atom and some aromatic rings, macrolide antibiotics can easily be converted from weak into potent MDR-reversing compounds. This finding also suggests that the molecular weight and lipophilicity of compounds have a crucial function in P-gp substrate specificity and/or inhibition, as earlier reported by Didziapetris *et al* (2003).

The Betti-modification endows TBN with higher cytotoxicity as compared with tylosin. In contrast to the Betti-base itself, this cytotoxic effect does not differ between parental and transfected L5178 cells, suggesting that the compound is not a substrate of the P-gp membrane pump. On the other hand, the acute toxicity of TBN seems to be low, as only 50 mg kg^–1^, but not 10 mg kg^–1^, given i.p. every second day over a period of 12 days resulted in limited weight loss. In this study, we further observed *in vivo* that even a single administration of a low dose of TBN (10 mg kg^–1^) restored completely the ability of tumours consisting of transfected L5178 cells to accumulate doxorubicin to the level of the parental doxorubicin-sensitive cell line. A certain time interval, for example 1 h after i.v. or 3 h after i.p. injections, between the administration of TBN and doxorubicin was required, as simultaneous co-administration of the compounds did not result in increased doxorubicin levels in resistant L5178 tumours. Consequently, we could also prove that TBN significantly increased the antitumour activity of doxorubicin on doxorubicin-resistant L5178 tumours. For instance, whereas 4 mg kg^–1^ doxorubicin given i.p. every second day did not result in any antitumoural effect on resistant L5178 tumours over a period of 12 days, the combination with 10 mg kg^–1^ TBN dramatically reduced the TV by 58% on day 12. This is in line with the results obtained when 4 mg kg^–1^ doxorubicin was given to mice bearing sensitive L5178 tumours: in this case a 46% reduction of TV was achieved. This data again imply that TBN is able to restore *in vivo* completely the sensitivity of transfected L5178 tumours towards doxorubicin.

Studies with *mdr1a* and *mdr1b* knock out mice have suggested that P-gp is not essential for basic physiological functions. However, as the P-gp membrane pump is expressed at the renal tubule and biliary and intestinal lumen, P-gp modulation can reduce the clearance of anticancer drugs, and by doing so, dramatically increase their toxicity (Sikic *et al*, 1997; Schinkel, 1998). Some inhibitors such as PSC 833 (Sikic *et al*, 1997) and biricodar (Rowinsky *et al*, 1998) have shown major pharmacokinetic interactions with anticancer drugs, whereas others such as XR9576 (Mistry *et al*, 2001) and LY 335979 (Dantzig *et al*, 1996) did not. Here, we show that TBN at the highest dose studied (50 mg kg^–1^) did not affect the clearance of doxorubicin from heart, liver, kidney and plasma, and hence the observed increased levels of the antitumoural compound in resistant L5178 tumours are not attributable to a sustained presence of the compound into the circulation.

In conclusion, the present observations state that the MDR reversal activity of the antibiotic tylosin can be dramatically increased by introducing a Betti-base in the basic macrolide structure. Our results indicate that TBN, the Betti-base derivative of tylosin, can chemosensitise resistant tumour cells to doxorubicin *in vitro* and *in vivo* by increasing the accumulation of doxorubicin. The co-administration of a low but efficacious dose of the compound with doxorubicin is well tolerated in mice. The lack of any influence of TBN on the pharmacokinetics of doxorubicin is a major argument for the specificity of the effect observed. Our results suggest that TBN is a potent modulator of the P-gp membrane pump and that the compound could be of clinical relevance to improve the efficacy of chemotherapy in MDR cancers. Nevertheless, future studies are needed to further characterise the interaction between TBN and P-gp, and possibly other ATP-binding cassette transporters, and long-term toxicity aspects of the compound.

## Figures and Tables

**Table 1:** FARs of the compounds calculated on the basis of the measured fluorescence values (see Materials and methods)

**Samples**	**Concentration (*μ*m)**	**FAR ± s.d.**
Verapamil	22	10±3.2
Betti-base	3.5	10±5.1
	35	8±1.7
Tylosin	3.5	1±0.0
	35	5±7.5
TBN	3.5	105±8.5
	35	94±25.5

Abbreviations: FAR=fluorescence activity ratio; TBN=*N*-tylosil-1-*α*-amino-(3-bromophenyl)-methyl-2-naphthol.

Non-resistant (parental cells) and human MDR1 gene-transfected L5178 mouse lymphoma cells were treated with rhodamine 123 and the individual compounds for 20 min at 37°C. The fluorescence of the cell population was then measured with a flow cytometer.

Mean and s.d. values were calculated from at least three independent experiments.

**Table 2:** IC values calculated for the investigated compounds

	**Transfected cells**			**Parental cells**		
**Compounds**	**IC_10_ *μ*M±s.d.**	**IC_50_ *μ*M±s.d.**	**IC_90_ *μ*M±s.d.**	**IC_10_ *μ*M±s.d.**	**IC_50_ *μ*M±s.d.**	**IC_90_ *μ*M±s.d.**
Betti-base	5.0±4.4	40.2±4.5	75.3±5.2	0.5±0.1	4.1±0.2	35.3±8.3
Tylosin	133.1±52.8	410.0±31.8	885.4±51.0	76.1±49.4	561.3±66.4	1046.3±182.4
TBN	11.1±9.2	41.4±6.8	55.2±2.5	9.4±4.4	40.3±2.7	71.3±1.3
Doxorubicin	0.02±0.01	1.8±0.5	14.6±1.7	0.005±0.001	0.022±0.2	0.359±0.05

Abbreviation: TBN=*N*-tylosil-1-*α*-amino-(3-bromophenyl)-methyl-2-naphthol.

Non-resistant (parental cells) and human MDR1 gene-transfected (transfected cells) L5178 mouse lymphoma cells were treated with different concentrations of the compounds or vehicle and incubated in cell culture plates at 37°C for 72 h. At the end of the incubation period, the relative cell density was determined using an MTT assay and optical density reading. From the inhibitory curves obtained, corresponding IC_10_, IC_50_ and IC_90_ values were calculated.

Mean and s.d. values were calculated from at least three independent experiments.

**Table 3:** IC_50_ values and AF calculated for doxorubicin, when combined with the Betti-base, tylosin or TBN

	**Transfected cells**		**Parental cells**	
	**IC_50_*μ*m ± s.e.**	**AF**	**IC_50_ *μ*m ± s.e.**	**AF**
Betti-base IC_10_	0.2±0.3	9.2	0.01±0.2	1.7
Betti-base IC_10/10_	2.5±0.5	0.7	0.02±0.2	1.0
Tylosin IC_10_	0.2±0.1	7.4	0.02±0.3	1.1
Tylosin IC_10/10_	0.2±0.1	7.4	0.02±0.2	0.6
TBN IC_10_	0.01±0.1	134.1	0.008±0.1	2.8
TBN IC_10/10_	0.2±0.1	10.5	0.05±0.02	0.4

Abbreviations: AF=antiproliferative factors; TBN=*N*-tylosil-1-*α*-amino-(3-bromophenyl)-methyl-2-naphthol.

Non-resistant (parental cells) and human MDR1 gene-transfected (transfected cells) L5178 mouse lymphoma cells were treated in cell culture plates at 37°C for 72 h with different concentrations of doxorubicin, and co-incubated with the IC_10_ or a 10-fold lower concentration (IC_10/10_) of the Betti-base, tylosin or TBN. At the end of the incubation period, the relative cell density was determined using an MTT assay and optical density reading. From the inhibitory curves obtained, corresponding IC_50_ values were calculated. AF were determined by dividing the IC_50_ value of doxorubicin in the absence of the Betti-base, tylosin or TBN by the IC_50_ value of doxorubicin in their presence.

Mean and s.e. values were calculated from at least four independent experiments.
